# Genome-wide CRISPR screen reveals novel host factors required for *Staphylococcus aureus* α-hemolysin-mediated toxicity

**DOI:** 10.1038/srep24242

**Published:** 2016-04-12

**Authors:** Sebastian Virreira Winter, Arturo Zychlinsky, Bart W. Bardoel

**Affiliations:** 1 Department of Cellular Microbiology Max Planck Institute for Infection Biology, Charitéplatz 1, 10117 Berlin, Germany

## Abstract

*Staphylococcus aureus* causes a wide variety of infections and antibiotic resistant strains are a major problem in hospitals. One of the best studied virulence factors of *S. aureus* is the pore-forming toxin alpha hemolysin (αHL) whose mechanism of action is incompletely understood. We performed a genome-wide loss-of-function screen using CRISPR/Cas9 technology to identify host targets required for αHL susceptibility in human myeloid cells. We found gRNAs for ten genes enriched after intoxication with αHL and focused on the top five hits. Besides a disintegrin and metalloproteinase domain-containing protein 10 (ADAM10), the host receptor for αHL, we identified three proteins, Sys1 golgi trafficking protein (SYS1), ADP-ribosylation factor 1 (ARFRP1), and tetraspanin-14 (TSPAN14) which regulate the presentation of ADAM10 on the plasma membrane post-translationally. Interestingly, we also showed that cells lacking sphingomyelin synthase 1 (SGMS1) resist αHL intoxication, but have only a slightly reduced ADAM10 surface expression. SGMS1 regulates lipid raft formation, suggesting that αHL requires these membrane microdomains for attachment and cytotoxicity.

*Staphylococcus aureus* infections range from mild skin to life–threatening, disseminated diseases. The recent rise of multidrug-resistant strains complicates effective treatment of these infections and is a major public health concern. *S. aureus* effectively disarms the innate immune system by exploiting several immune evasion strategies^1^. Alpha hemolysin (αHL) is an important *S. aureus* toxin and is essential for virulence in different infection models^2^,^3^,^4^.

αHL oligomerizes and forms pores on the host cell plasma membrane of a wide variety of cells. It can either trigger cell death or modulate several cellular processes, including cytokine production and proliferation^2^. αHL interacts with A Disintegrin And Metalloproteinase domain-containing protein 10 (ADAM10), which serves as its receptor^5^. ADAM10 is a transmembrane protease, shedding numerous targets such as TNF-α, HB-EGF or Notch, thereby modifying cellular signaling^6^. Most of ADAM10’s substrates are shared with other ADAM family members and ADAM10 expression in different cells correlates with susceptibility to αHL. Indeed, αHL binding increases ADAM10’s protease activity, which disrupts the epithelial barrier function and leads to lethal lung injury, as shown via ADAM10 conditional knockouts in the lung^7^.

Clustered regularly interspaced short palindromic repeats (CRISPR) and Cas genes form a prokaryotic immune system that is encoded in many bacteria and archaea^8^. The CRISPR/Cas9 system from *Streptococcus* is now used for targeted disruption and modification of genes in human cells^9^. Cas9 is a nuclease that can be directed to the sequence of interest by a guidance RNA (gRNA) sequence to create double strand breaks in the DNA. This induces activation of the host repair mechanisms, often resulting in improper repair of the lesion site and disruption of the target gene. Recently, synthesis of a CRISPR library containing 120,000 gRNAs targeting more than 19,000 human genes paved the way for genome-wide screening in human cells^10^.

In this study, we used this genome-wide screening approach to identify host factors required for αHL cytotoxicity. Lately, it has been reported that myeloid-specific effects of αHL are important determinants for the outcome of infection^11^. Therefore, we chose the human myeloid cell line U937 for our experiments. We transduced these cells with a CRISPR library targeting 19,050 human genes and 1864 miRNAs and intoxicated with αHL to obtain survivors that contain a mutation in genes essential for αHL-mediated cell death. Sequencing revealed the enrichment of genes that are involved in αHL toxicity including ADAM10. Single-cell cloning and complementation confirmed that all further analyzed hits from the screen are required for αHL-mediated cell death. Three genes regulate ADAM10 post-translationally and their deficiency reduces ADAM10 levels on the surface, thereby decreasing toxin binding. The importance of lipid rafts is underlined by the requirement of sphingomyelin synthase 1 (SGMS1) to allow binding of αHL to the host cell.

## Results

### Genome-wide CRISPR screen reveals novel host factors required for αHL susceptibility

**Figure 1:**
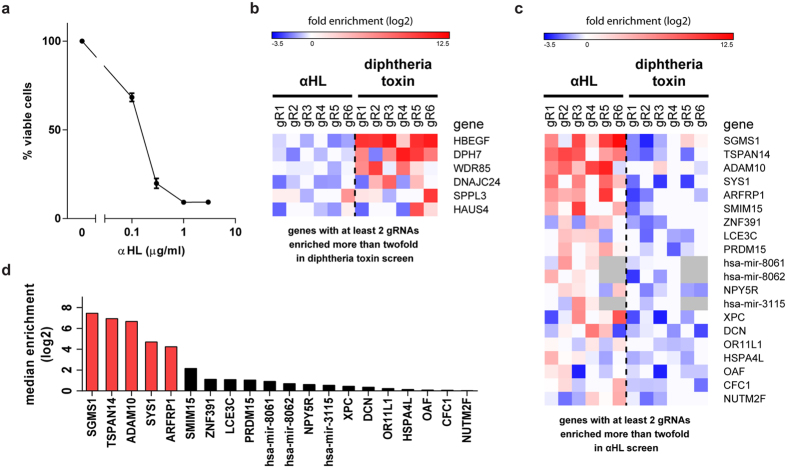
Genome-wide CRISPR screen identifies novel αHL host targets. (**a**) U937 cells were incubated with various concentrations of αHL overnight and the percentage of viable cells was analyzed by flow cytometry. Data represent mean values of three independent experiments + SD. (**b,c**) Individual enrichment (log2) of gRNAs is shown for the most enriched genes following intoxication with DT (**b**) or αHL (**c**). Ranking is based on the median enrichment (log2) among all six gRNAs present in the library. (**d**) Median enrichment of six gRNAs for the 20 most enriched genes in the αHL screen is shown .

To identify host genes required for αHL toxicity, we used a genome-wide CRISPR library generated by the Zhang laboratory^10^,^12^. This library contains over 120,000 gRNAs with 6 gRNAs per gene for 19,050 human genes and 4 gRNAs per miRNA. We first determined the toxicity of αHL on the human myeloid cell line U937 via flow cytometry by gating for DRAQ7-negative (a membrane-impermeable dye) cells with a forward and side scatter comparable to untreated cells (Supplementary Fig. S1). More than 85% of U937 cells were killed by 0.5 μg/ml of αHL overnight and we used this dose to perform the screen (Fig. 1a). We transduced U937 cells with the CRISPR library at an MOI of 0.3–0.5 and selected for cells with stable viral integration. Subsequently, we intoxicated the cells with αHL or diphtheria toxin (DT), as control, for two weeks to allow outgrowth of resistant mutants. We isolated the genomic DNA of the survivors, amplified the gRNA sequences by PCR and analyzed by high-throughput sequencing which gRNAs are present in the toxin-resistant cells (Supplementary Fig. S2).

The targets of DT, which blocks translation and therefore has a different mechanism than αHL, were well characterized by genome-wide screening approaches^13^,^14^ and served as a control in our experiments. Cells transduced with the CRISPR library and cultured without toxins for two weeks contained 88% of all gRNAs present in the library, whereas selection with αHL or DT decreased the amount of identified gRNAs below 30%. The number of reads per gRNA, expressed as fold of the median read number, ranged from 0.1–28 (unselected cells) to 0.2–15569 (αHL) and 0.2–57965 (DT), showing that intoxication with αHL or DT enriches a small portion of the gRNAs. For further analysis, we calculated the enrichment of each gRNA after intoxication compared to the untreated CRISPR library-transduced cells. The enrichment of multiple gRNAs per gene is the best indicator of a valid hit. As expected, we identified *HBEGF*, *WDR85* and factors involved in diphthamide synthesis (*DPH7* and *DNAJC24*) as genes required for susceptibility to DT (Fig. 1b)^13^. We found at least three gRNAs enriched twofold (log2 enrichment compared to unselected cells >1) for 5 genes following DT intoxication. Selection with αHL resulted in ten genes with at least three gRNAs enriched (Supplementary Table 1). As the top hits, we identified the receptor ADAM10, which is known to bind the toxin, and four additional genes: Sys1 golgi trafficking protein (*SYS1*), ADP-ribosylation factor 1 (*ARFRP1*), tetraspanin-14 (*TSPAN14*) and sphingomyelin synthase 1 (*SGMS1*) (Fig. 1c,d). For these five genes, at least four out of six gRNAs were enriched more than nine fold and we therefore selected them for further analyses.

### Cells targeted for screening hits are resistant to αHL

To validate these five genes, we selected two of the highly enriched gRNAs to target each hit in U937 cells. As a control, we used either a scrambled 20 nucleotide gRNA or a gRNA targeting neutrophil elastase, a gene unlikely to be involved in αHL-mediated toxicity, and referred to as “control gene” in this report. Cells transduced with gRNAs against any of the candidates, but not the controls, became resistant to αHL (Supplementary Fig. S3). This confirms that the screen identified genes involved in αHL toxicity and that the enrichment cut-off to select candidates for further analyses was stringent.

**Figure 2:**
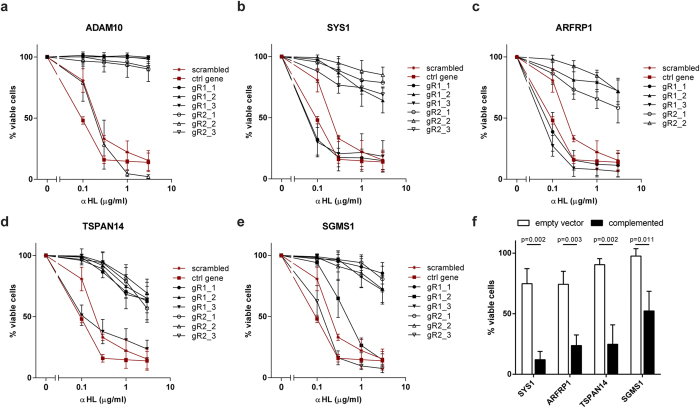
Individual targeting of the αHL hits results in αHL-resistant cells. (**a–e**) U937 clones derived from single cells by limiting dilution for each of the two gRNAs targeting (**a**) ADAM10, (**b**) *SYS1*, (**c**) *ARFRP1*, (**d**) *TSPAN14* or (**e**) *SGMS1* were incubated with 1 μg/ml of αHL overnight. Percentage of viable cells was determined by flow cytometry and gating for DRAQ7-negative and forward side scatter characteristics as control U937 cells. As a control, a random scrambled and a gRNA targeting an unrelated control gene (neutrophil elastase, NE) were included. (**f**) For each target gene, a CRISPR-targeted clone was complemented with a copy of the gene, modified via silent mutations to avoid editing by Cas9. Transduction with an empty vector served as a control. Cells were stimulated with 0.3 μg/ml αHL and after 40 hours the percentage of viable cells was measured by flow cytometry. All graphs (**a**–**f**) represent mean + SD of three independent experiments. (**f**) Statistical testing was performed using t test and p values are indicated.

The CRISPR/Cas9 system introduces double-strand breaks (DSBs) efficiently, although a successful disruption of the target gene only occurs if a frameshift mutation is introduced by DNA repair mechanisms in both alleles. Transduction with an individual gRNA produces a heterogeneous population of cells where some cells have either no mutation or inframe mutations that still encode a functional protein. This “untargeted” population can explain the incomplete resistance and differences in survival between targeted genes in pooled populations (Supplementary Fig. S3). Furthermore, the difference in efficiency using different gRNAs has been observed in other CRISPR/Cas9 screens^14^. To avoid this heterogeneity, we obtained single cell clones by limiting dilution. As expected, we isolated both toxin-resistant and -susceptible clones (Fig. 2a–e). Notably, we observed that the frequency of frameshift mutations introduced varies between gRNAs with ADAM10 gR1 being the most efficient as we did not obtain any toxin-susceptible clones. In contrast, we identified both toxin-resistant and -susceptible clones using ADAM10 gR2 (Fig. 2a and Supplementary Fig. S4). Successful targeting of *SYS1*, *ARFRP1*, *TSPAN14* and *SGMS1* occurred in around 50% of the clones, which correlates with the results obtained with mixed cell populations (Supplementary Fig. S3). In contrast to cells lacking ADAM10, around 20% of *SYS1*-, *ARFRP1*-, *TSPAN14*-, and *SGMS1*-targeted cells are susceptible to a high αHL concentration of 3 μg/ml.

To rule out that the toxin-resistant clones resulted from off-target mutations, we complemented the clones by overexpressing CRISPR-resistant genes. We introduced silent mutations in regions targeted by the gRNAs to avoid disruption of the overexpression constructs by CRISPR/Cas9. Lentiviral complementation of *SYS1*-, *ARFRP1*-, *TSPAN14*- and *SGMS1*-targeted cells restored susceptibility to αHL (Fig. 2f). However, none of the identified hits conferred resistance to streptolysin O (SLO), a pore-forming toxin of group A, C and G streptococci (Supplementary Fig. S5). These results confirm that the genes we identified in the genome-wide CRISPR screen are required for αHL toxicity.

### Screening hits are essential for αHL binding to cells

TSPAN14 was previously associated with ADAM10 surface expression^15^,^16^. SYS1 and ARFRP1 interact with each other and play a role in protein trafficking in the Golgi apparatus^17^, although there is no known link with either ADAM10 or αHL susceptibility. SGMS1 synthesizes sphingomyelin, a major constituent of the plasma membrane, and is involved in the formation of lipid rafts^18^. Cells lacking SGMS1 show reduced sphingomyelin levels on their plasma membrane and are resistant to cytolethal distending toxins (CDTs), suggesting that, like for CDTs, lipid rafts are required for αHL-mediated toxicity^13^.

**Figure 3:**
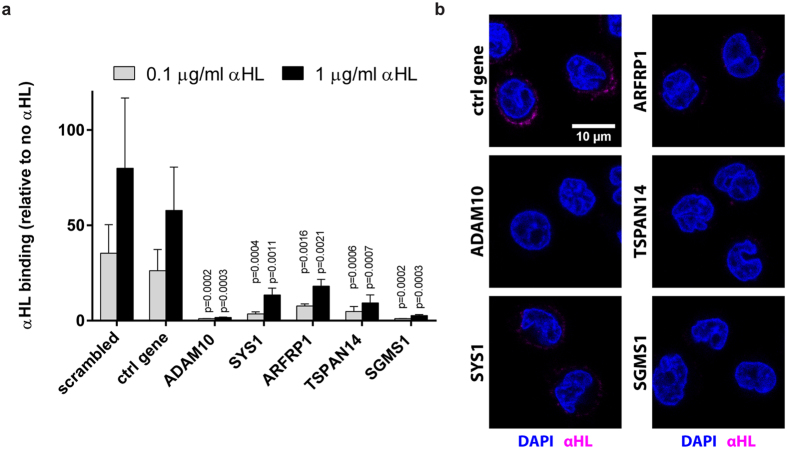
αHL binding is impaired in toxin-resistant CRISPR-targeted cells. (**a**) U937 cells were incubated with 0–1 μg/ml fluorescently labeled αHL for 30 minutes at 37 °C. For each gene we tested one αHL-resistant clone. Binding was analyzed by gating for DRAQ7-negative cells using flow cytometry and expressed relative to 0 μg/ml αHL. Graphs indicate mean + SD of three independent experiments and statistical testing was performed using unpaired one-way ANOVA with correction for multiple comparisons (Dunnett). (**b**) U937 clones were challenged with 2 μg/ml αHL-Cys-His-Alexa647 for 5 minutes, fixed and stained with DAPI. Images were obtained by confocal microscopy. Nuclei (blue) and cell-bound αHL-Cys-His-Alexa647 (magenta) are shown. These are representative confocal z-planes of two independent experiments.

To address if these genes are required for αHL binding, we incubated cells with fluorescently labeled αHL. We introduced a cysteine at the C-terminus of αHL, which does not contain any endogenous cysteine residues, to allow site-specific labeling with the fluorescent dye Alexa Fluor 647 (Supplementary Fig. S6). The fluorescent αHL bound to control cells but not to cells with a gRNA targeting ADAM10 (Fig. 3a), which correlates with previous findings^5^. Disruption of *SYS1*, *ARFRP1*, *TSPAN14* or *SGMS1* decreased αHL binding to U937 cells. Analysis by live cell microscopy revealed that αHL binds to the host cell within minutes (Supplementary Video 1). Furthermore, confocal imaging confirmed that targeting any of the candidate genes prevents binding of αHL to the cell surface (Fig. 3b). These experiments demonstrate that the selected hits of our screen impair αHL activity by preventing binding of the toxin to the cells.

### SYS1, ARFRP1 and TSPAN14 regulate ADAM10 surface expression

**Figure 4:**
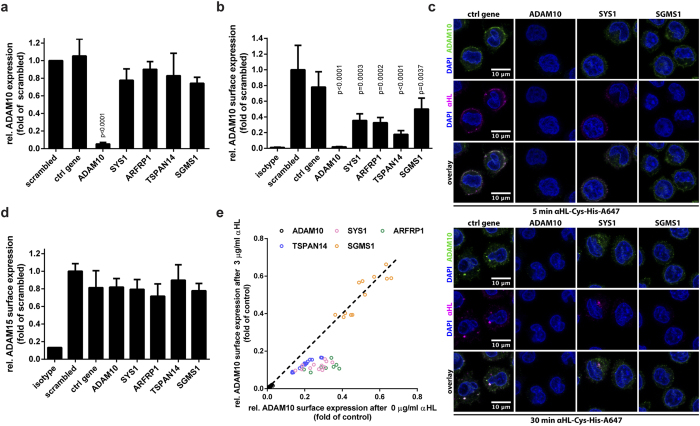
Surface ADAM10 regulates susceptibility to αHL. (**a**) CRISPR-targeted U937 cells were analyzed for gene expression of ADAM10, quantified by qPCR and expressed as relative value compared to the RPL32 gene. (**b**) U937 clones targeted with the gRNAs to the indicated genes were incubated with an ADAM10 antibody, followed by a PE-labeled secondary goat-anti-mouse antibody. Geometric mean of ADAM10-PE staining was analyzed by flow cytometry. Values represent the relative ADAM10 surface expression compared to control cells (WT U937 or cells transduced with scrambled gRNA). (**c**) U937 clones were intoxicated with 2 μg/ml αHL-Cys-His-Alexa647 (magenta) for the indicated times, fixed, permeabilized and ADAM10 was visualized by staining with an anti-ADAM10 antibody (green). Nuclei were stained with DAPI (blue). Shown are representative confocal z-planes of two independent experiments. (**d**) U937 CRISPR clones were stained with anti-ADAM15 and analyzed by flow cytometry. Data are expressed as fold expression relative to scrambled control. (**e**) U937 CRISPR clones were incubated with either 0 or 3 μg/ml αHL overnight at 37 °C. Subsequently, surface expression of ADAM10 was analyzed by flow cytometry. Dots represent individual experiments of αHL resistant CRISPR clones. Data are plotted as relative ADAM10 surface expression compared to WT U937 or scrambled at 0 versus 3 μg/ml αHL. (**a**,**b**,**d**) Shown are mean + SD of three independent experiments and statistical testing was performed using unpaired one-way ANOVA with correction for multiple comparisons (Dunnett). (**c**) representative images of two independent experiments.

Decreased binding of αHL to the cells suggests that all hits identified in the screen interact directly with the toxin or influence factors involved in toxin-cell interaction. The only proposed αHL-interacting protein is ADAM10 and cells lacking this receptor are resistant to αHL^5^. To analyze if there was a decrease in ADAM10 expression in cells lacking SYS1, ARFRP1, TSPAN14, or SGSM1, we quantified ADAM10 transcription by qRT-PCR (Fig. 4a). None of these proteins regulate the transcription of ADAM10.

Since αHL interacts with ADAM10 on the cell surface^5^, we asked whether any of the candidate genes affects ADAM10 surface localization. We stained CRISPR-targeted cells with an antibody directed against the ectodomain of ADAM10. As expected, ADAM10 was not detected at the surface of ADAM10-targeted clones; the mean fluorescence intensity values were similar to an isotype control antibody (Fig. 4b and Supplementary Fig. S7). Notably, we also observed decreased surface levels of ADAM10 when the other genes were targeted: 60% reduction in *SYS1* and *ARFRP1* mutants, 80% in *TSPAN14* mutants and 50% in *SGMS1* mutants. In addition, confocal microscopy confirmed that targeting *SYS1*, *ARFRP1*, *TSPAN14*, and *SGMS1* decreased ADAM10 surface staining and binding of αHL to cells (Fig. 4c and Supplementary Fig. S8). In control cells, αHL and ADAM10 colocalize at the plasma membrane after 5 minutes, whereas prolonged incubation results in their intracellular colocalization. These results suggest that deficiency in ARFRP1, SYS1, and TSPAN14 causes resistance to αHL at least partially because of reduced surface ADAM10 expression. Indeed, the partial susceptibility to high concentration of αHL when these three genes are mutated (Fig. 2a–e) might be explained by the residual expression of ADAM10 on the surface. This is consistent with the observation that *SYS1*-, *ARFRP1*- and *TSPAN14*-targeted cells bind and internalize low levels of αHL (Fig. 4c and Supplementary Fig. S8).

To test if targeting of the Golgi-resident proteins, SYS1 and ARFRP1, causes a common defect in protein trafficking to the surface we determined the levels of ADAM15, a closely related ADAM family member, on the cell surface of U937 cells (Supplementary Fig. S9). Targeting any of the five genes did not affect the surface expression of ADAM15, indicating that neither SYS1 nor ARFRP1 are general regulators of the localization of the ADAM protease family (Fig. 4d).

Targeting of *SGMS1* reduces αHL toxicity strongly, although ADAM10 surface expression remains relatively high compared to the other targeted genes (Fig. 4b and Supplementary Fig. S7). In *SYS1*-, *ARFRP1*- or *TSPAN14*-targeted cells, treatment with αHL reduces subsequent ADAM10 surface staining with an anti-ADAM10 antibody. This is in line with our findings that these cells bind low levels of αHL and internalize it together with ADAM10. In contrast, labeling with an anti-ADAM10 antibody in *SGMS1*-targeted cells is unaffected even after incubation with 3 μg/ml of αHL (Fig. 4e). To check if ADAM10 processing in the Golgi is affected in any of the CRISPR-targeted clones, we performed western blot analysis of ADAM10 (Supplementary Fig. S10). ADAM10 was not detectable in ADAM10-targeted cells and targeting of *SYS1*, *ARFRP1* and *TSPAN14* led to intracellular accumulation of the unprocessed, immature form of ADAM10, while targeting of *SGMS1* did not affect ADAM10 maturation. These findings indicate that *SGMS1*-targeted cells display reduced αHL binding and toxicity independent of ADAM10 surface expression levels.

## Discussion

The clinically relevant pathogen *S. aureus* secretes the pore forming toxin αHL as one of its most important virulence factors. We screened for genes required for the toxic activity of αHL in myeloid cells, which have recently been shown to be relevant target cells of αHL^11^, by employing the CRISPR/Cas9 technology. To control for the specificity of our screen, we treated cells with either DT or αHL. DT inhibits protein synthesis while αHL is a pore-forming toxin. As expected, the αHL screen identified different genes when compared to the diphtheria toxin screen, which was validated by identifying genes already known to be essential for diphtheria toxicity^13^,^14^. More importantly, in the αHL screen we confirmed that ADAM10 is the major target of the toxin as proposed by biochemical interaction and functional studies^5^. The screen identified ten genes for which at least three gRNAs were enriched and which have not previously been linked to αHL toxicity. During further analyses we found *SYS1*, *ARFRP1* and *TSPAN14* to be important for ADAM10 expression on the cell surface. We also identified *SGMS1*, which is required for αHL toxicity, but affects ADAM10 expression only moderately. All these genes are not required for the toxicity of the pore-forming toxin SLO, indicating that pore-forming toxins target host cells in diverse ways. Surprisingly, SGMS1-deficient cells were even more susceptible to SLO. This highlights the importance of plasma membrane lipids as determinants for toxin interactions with host cells. To further explore if there are more than the five hits among the ten identified candidates, we targeted *SMIM15* and *PRDM15* with three gRNAs each and found that they exhibit increased resistance to αHL compared to control cells (Supplementary Fig. S11). Interestingly, genes associated with the Nlrp3 inflammasome were not identified in our screen, even though it has been reported that αHL can active Nlrp3 in monocytic cells^19^,^20^. This might be explained by differences in the cells used since murine bone marrow-derived macrophages also do not activate Nlrp3 in response to recombinant αHL and it is not clear whether αHL activates Nlrp3 directly or via substances released from intoxicated cells. Also other genes that have been linked previously to αHL susceptibility, including apoptotic caspases, were not identified in this screen^21^,^22^,^23^,^24^. Possible reasons are pathway redundancy and the stringent selection for survivors with αHL for two weeks, biasing against non-essential modulators of αHL intoxication. In conclusion, additional host targets of αHL may exist, since genetic screens fail to identify redundant or essential genes and our study is limited to U937 cells.

Several members of the tetraspanin (TSPAN) family, including TSPAN14, play an important role in maturation and trafficking of ADAM10^15^. Our data indicate that TSPAN14 is the dominant TSPAN regulating ADAM10 surface expression in U937 cells. Even though gRNAs targeting other TSPANs were included in the GeCKO library, no other TSPAN had at least two gRNAs enriched after αHL treatment, suggesting that other TSPANs have no function in regulating ADAM10 or are not expressed in U937 cells. SYS1 and ARFRP1 interact and form a protein complex^17^. SYS1 targets ARFRP1 to the Golgi apparatus, where it interacts with proteins that coordinate the Golgi function. The relevance of these proteins in neither ADAM10 surface expression nor αHL toxicity was known. ADAM10 has a Golgi retention signal and is mostly found in its inactive form within this organelle^25^. Our data demonstrate that SYS1 and ARFRP1 regulate trafficking of ADAM10 to the surface in its active form. Interestingly, our results suggest that SYS1 and ARFRP1 are not general regulators of protein trafficking since levels of ADAM15, which is closely related to ADAM10, were unaffected in cells where SYS1 or ARFRP1 were not functional. Together these data highlight that ADAM10 delivery to the plasma membrane has a dedicated control pathway.

SGMS1 is required for toxic activity of CDTs, which are AB toxins with DNase activity^13^. Although the mechanism of action of CDTs differs from αHL, both toxins require binding to a receptor expressed on the plasma membrane. SGMS1 regulates the sphingomyelin content in the outer leaflet of the plasma membrane, which is involved in lipid raft formation^18^. Several studies showed that clustered sphingomyelin-cholesterol domains are required for pore formation by αHL^26^,^27^. Furthermore, αHL induces ADAM10 cluster formation and subsequent relocalization to lipid rafts^5^,^28^. Affecting lipid rafts by targeting SGMS1 may explain the αHL-resistant phenotype of cells without extensive downregulation of ADAM10 surface expression.

The exact mechanism by which ADAM10 is involved in αHL toxicity is still unclear. Outcomes of αHL intoxication depend on the dose and the cell type. Low concentrations of toxin induce apoptosis, whereas higher concentrations cause unspecific, ADAM10-independent, binding and killing^5^,^29^. We used a low concentration of αHL, sufficient to kill most cells after overnight incubation whilst avoiding unspecific binding and killing of the cells. In addition to the requirement of ADAM10 for αHL binding, it has also been shown that αHL increases the proteolytic activity of ADAM10 triggering tissue damage^30^. Our observations that both ADAM10- and *SGMS1*-targeted cells allow no αHL binding or αHL-induced ADAM10 uptake favor the model of a cooperative interaction of αHL with a proteinaceous receptor, ADAM10, in combination with lipids such as sphingomyelin^2^.

Recently, Popov *et al*. published an elegant screen for αHL using mutagenized human haploid cells^31^. These investigators also identified ADAM10 as a required gene and showed that PLEKHA7 and other junctional components were also necessary for αHL toxicity. Interestingly, they picked up TSPAN33, suggesting that different members of the TSPAN family are required in different cells for ADAM10 presentation on the cell surface. The difference between the two screens may be due to both the methodology and the origin of cell. It will certainly be interesting to test whether the genes found in both screens have a function in other cells, although myeloid cells do not have junctions. It would be interesting to show the function of these genes *in vivo*, however the contribution of the identified genes in our screen is difficult to address *in vivo* since knockouts for all the identified genes are embryonic lethal in mice. More importantly, the results obtained by Popov *et al*. and our data likely point to the difference in αHL toxicity functions between diverse target cells and might explain the diverse pathogenesis of *S. aureus*.

The identification of genes required for proper ADAM10 trafficking is of great interest since this protease is also relevant in Alzheimer’s disease (AD)^32^,^33^. Aggregation of the amyloidβ peptide causes amyloid plaque formation in the brain which is a hallmark of AD^34^. ADAM10 processes the amyloidβ precursor preventing its aggregation and amyloid plaque formation^25^. Boosting ADAM10 activity has potential therapeutic relevance^33^. Since the identified hits in this study target yet unidentified players in ADAM10 regulation they may provide novel avenues to understand the role of ADAM10 in AD.

αHL plays a crucial role in *S. aureus* infections as illustrated by different infection models^3^,^4^. We, like Popov *et al*., find in an unbiased screen that ADAM10 is the main target of αHL. Additional genes identified in our screen underline this, showing an important role in the regulation of ADAM10 expression. The requirement of SGMS1 illustrates the importance of membrane composition for αHL-mediated cytotoxicity. These findings help to further improve our understanding of the molecular pathogenesis of *S. aureus* infections.

## Materials and Methods

### Cells and reagents

U937 and HEK293T cells were obtained from ATCC and DSMZ, respectively. Cells were propagated in RPMI or DMEM, supplemented with 10% FCS, glutamine and penicillin/streptomycin. ViraPower packaging mix (Thermo Fisher Scientific) was used to produce pseudotyped lentivirus. αHL and diphtheria toxin were obtained from Sigma-Aldrich, SLO was purchased from AbD Serotec. Mouse IgG2b against the ectodomain of human ADAM10, mouse IgG1 against human ADAM15 and isotype controls were from R&D Systems. Mouse IgG1 against human ADAM10 (clone 11G2) was from abcam and rabbit IgG against human GAPDH (clone 14C10) was from Cell Signaling Technology. DRAQ7 was purchased from BioStatus. Alexa Fluor 647 C_2_ Maleimide and DAPI were from Thermo Fisher Scientific and PE-labeled goat anti-mouse IgG was from BioLegend. Human GeCKO v2 Library (Addgene #1000000048) and lentiCRISPR v2 (Addgene plasmid # 52961) were obtained from Addgene.

### CRISPR screen

The human GeCKO v2 library containing 120,000 gRNA sequences in the lentiCRISPR v2 vector containing Cas9 was transformed into *E. coli* as described^12^. Briefly, 293T cells were transfected with isolated plasmid pool of the GeCKO v2 library and ViraPower packaging plasmids for lentivirus production. Lentivirus was harvested and concentrated by centrifugation before U937 cells were transduced. After two days, 2.5 μg/ml puromycin was added to select for transduced cells. Surviving cells were incubated with 0.5 μg/ml αHL or 10 ng/ml diphtheria toxin 7 days after transduction for two weeks. The unselected library was also kept in culture before isolating the DNA. A 1^st^ PCR was performed with primers 5′-AATGGACTATCATATGCTTACCGTAACTTGAAAGTATTTCG-3′ and 5′-TCTACTATTCTTTCCCCTGCACTGTTGTGGGCGATGTGCGCTCTG-3′ annealing up- and downstream of the gRNA sequence. The PCR product was purified and a 2^nd^ PCR was performed with primers 5′-TCTTGTGGAAAGGACGAAACACCG-3′ and 5′-TCTACTATTCTTTCCCCTGCACTGT-3′ annealing at the sequence that was introduced using the primers of the 1^st^ reaction. The PCR product containing gRNA sequences that were integrated into the genomes of the cells were sequenced by parallel sequencing using a HiSeq 2000 (Illumina). Reads for each gRNA were normalized to counts per million total reads and all counts below 5 were set to 5 per million reads to remove noise of low read counts and avoid that gRNAs appear enriched when all reads are below 5 and hence within noise. Enrichment of each individual gRNA was calculated compared to the unselected U937 cells and expressed as log2 enrichment. Enrichment data were plotted as a heatmap using the GENE-E tool (http://www.broadinstitute.org/cancer/software/GENE-E/index.html).

### Complementation

Sequences of the genes *SYS1, ARFRP1, TSPAN14*, and *SGMS1* were modified in the 20 nucleotide CRISPR region and PAM sequence for all six gRNAs present in the CRISPR library with at least four mutations without changing the amino acid sequence. The modified sequences were synthesized and cloned into pLenti6/V5-DEST (Thermo Fisher Scientific). The pLenti6/V5-DEST with genes of interest or the empty vector were used to generate lentivirus to transduce U937 cells. After transduction, cells were selected with 10 μg/ml blasticidin.

### Flow cytometry

Cells were challenged with the indicated concentrations (0–3 μg/ml) of αHL for 20–40 hours. The cells were centrifuged and resuspended in 100 μl RPMI + 0.5% HSA containing 0.5 μg/ml anti-ADAM10/anti-ADAM15 antibody or the corresponding isotype control. After incubation for 30 minutes at 4 °C, cells were washed and incubated with PE labeled goat anti-mouse IgG as secondary antibody and 0.6 μM DRAQ7 for 30 minutes at 4 °C. To determine susceptibility to SLO, cells were challenged with 0–10 μg/ml of SLO for six hours, washed with PBS and resuspended in 0.6 μM DRAQ7 in 0.5% FCS/PBS. After washing cells, the staining was analyzed using a MACSQuant Analyzer 10 flow cytometer and data were analyzed by FlowJo v10. The percentage of viable cells was determined by gating DRAQ7-negative cells with an unaffected forward and side scatter compared to untreated cells.

To analyze αHL binding, cells were resuspended in 100 μl RPMI with 0.5% HSA and then intoxicated with varying concentrations of αHL-Cys-His-Alexa647 for 30 minutes at 37 °C. Cells were put on ice, washed and cells with a disrupted membrane integrity were marked with 2.5 μg/ml DAPI and excluded from the analysis. Binding of αHL was determined by quantifying Alexa Fluor 647 fluorescence by flow cytometry.

### qPCR

Total RNA was isolated from CRISPR-targeted cells with TRIzol, according to the manufacturer’s protocol. cDNA was synthesized from purified RNA using QuantiTect Reverse Transcription kit from Qiagen. qPCR was performed using SYBR Green PCR Master Mix (Thermo Fisher Scientific) and ADAM10 primers (GTGTACGTGTGCCAGTTCTGATG & CTGAAGTGCCTACTCCACTGC) or RPL32 primers (CATCTCCTTCTCGGCATCA & AACCCTGTTGTCAATGCCTC) as a control.

### Microscopy

Cells were intoxicated with αHL-Cys-His-Alexa647 in RPMI, supplemented with 10% FCS, 2 mM glutamine and penicillin/streptomycin. After the indicated times, cells were fixed with a final concentration of 2% paraformaldehyde. Cells were washed after 10 minutes with PBS, permeabilized with 0.1% saponin for 10 minutes and blocked with 1% BSA/PBS for 15 minutes. To stain ADAM10, cells were incubated with 2 μg/ml anti-ADAM10 (11G2) for 2 hours at room temperature. After washing in PBS, cells were incubated with 4 μg/ml anti-mouse IgG-Alexa488 antibody for 1 hour at room temperature. Finally, cells were washed several times in PBS, incubated for 5 minutes with 2.5 μg/ml DAPI, washed again and imaged. Colocalization analysis was performed on entire Z-stacks using ImageJ and the Coloc 2 plugin with the Costes significance test (100 iterations).

For live cell imaging, cells were resuspended in Live Cell Imaging Solution (Thermo Fisher Scientific), supplemented with 10% FCS, 2 mM glutamine, 1x MEM vitamin solution, 4 g/L glucose and penicillin/streptomycin. Imaging was performed at 37 °C using a Leica TCS SP8 confocal microscope. Cells were then intoxicated with 2 μg/ml αHL-Cys-His-Alexa647 10 minutes after acquisition was started.

### Expression and labeling of αHL

The αHL gene was synthesized and modified by adding a C-terminal cysteine followed by a thrombin cleavage site and a His-Tag. The modified gene was cloned into pET28a, transformed into *E.coli* BL21 and induced with 0.5 mM IPTG. His-tagged αHL was purified using HisTrap columns (GE Healthcare) according to the manufacturer’s protocol. Purified αHL was concentrated to 10 mg/ml and incubated with 10x molar excess of Alexa Fluor 647 Maleimide for 2 h at room temperature. Excess dye was removed via HiTrap desalting columns (GE Healthcare).

### Western blot

U937 cells were counted, washed twice with PBS and lysed in non-reducing Laemmli buffer. Two million cells were loaded per lane on a non-reducing SDS-PAGE gel. Gel was blotted on nitrocellulose membrane and ADAM10 was detected with the monoclonal mouse anti-ADAM10 (11G2) antibody. As a loading control, GAPDH was detected via rabbit anti-GAPDH (14C10) antibody. Densitometric analysis to estimate the relative amount of processed ADAM10 was performed with ImageJ.

## Additional Information

**How to cite this article**: Virreira Winter, S. *et al*. Genome-wide CRISPR screen reveals novel host factors required for *Staphylococcus aureus* α-hemolysin-mediated toxicity. *Sci. Rep*. **6**, 24242; doi: 10.1038/srep24242 (2016).

## Supplementary Material

Supplementary Information

Supplementary table S1

Supplementary Video S1
