# Supplementary material for: Genome-wide CRISPR screen reveals novel host factors required for Staphylococcus aureus α-hemolysin-mediated toxicity
Source: Sci Rep. 2016 Apr 12;6:24242. doi: 10.1038/srep24242 (PMC4828653; doi:10.1038/srep24242)
Supplement: Supplementary Information [file srep24242-s1.pdf]

Genome-wide CRISPR screen reveals novel host factors required for *Staphylococcus aureus* α-hemolysin-mediated toxicity

Sebastian Virreira Winter, Arturo Zychlinsky and Bart W. Bardoel

Department of Cellular Microbiology
Max Planck Institute for Infection Biology, Charitéplatz 1, 10117 Berlin, Germany

Supplementary Figure Legends

Supplementary Table 1. enrichment (log2) of gRNAs compared to unselected U937 cells

gRNAs targeting the same gene were randomly numbered from 1-6. Enrichment (log2) of each individual gRNA was calculated compared to the unselected U937 cells.

Supplementary Video 1. αHL binds cells within minutes and is internalized at later time points

Ctrl gene-targeted cells were intoxicated with 2 µg/ml αHL-Cys-His-Alexa647 10 minutes after acquisition was started. Left: Fluorescence signal of αHL-Cys-His-Alexa647 (red). Right: Brightfield image of the corresponding field of view.

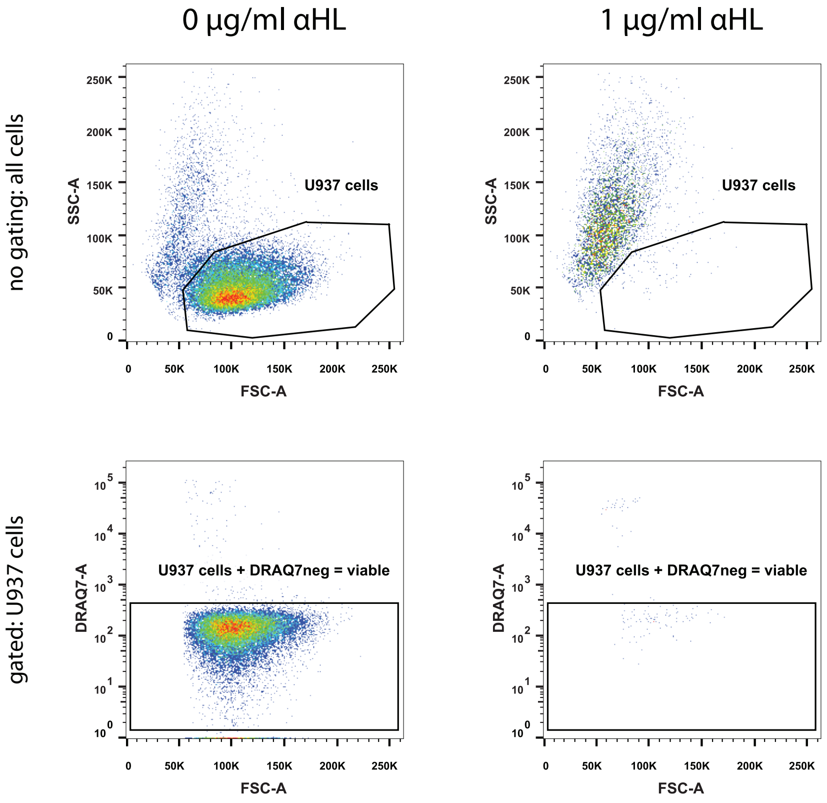

Supplementary Figure S1. Gating of viable cells

U937 cells were treated with 1 µg/ml αHL for overnight. Cells were stained with DRAQ7 and analyzed by flow cytometry. Viable cells were gated by forward and side scatter profile followed by gating for DRAQ7-negative cells.

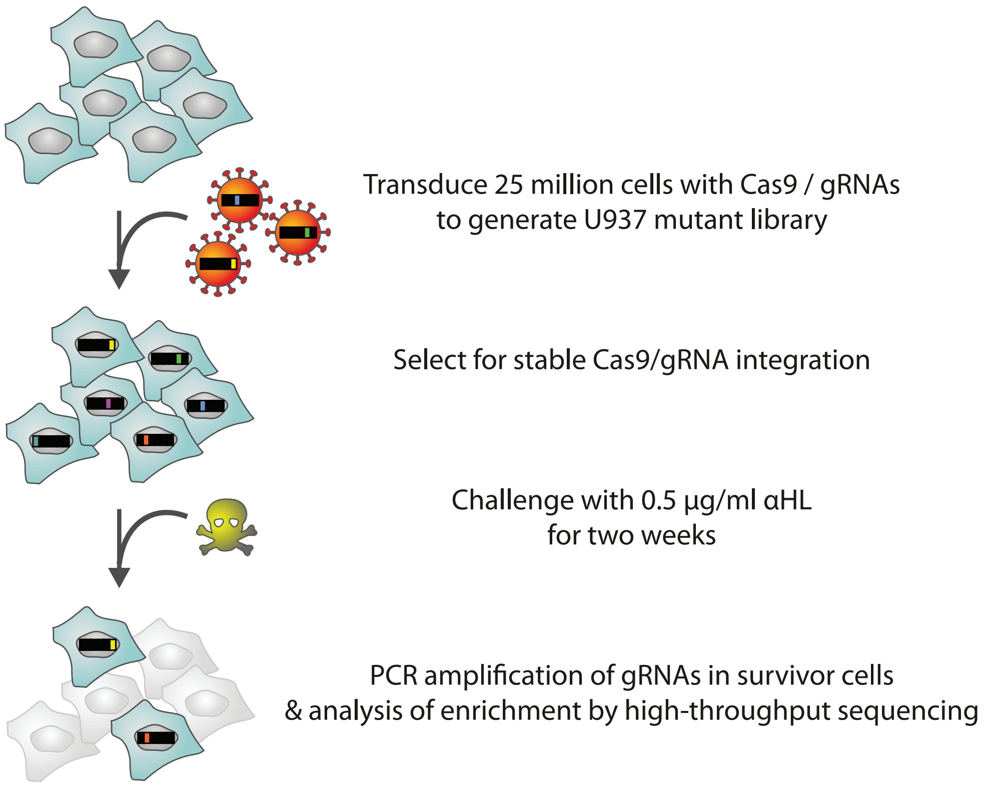

Supplementary Figure S2. CRISPR screen approach

25 million cells were transduced with a lentiviral CRISPR library containing 120,000 different gRNAs at an MOI of 0.3-0.5. The cells were selected for stable viral integration with 2.5 µg/ml puromycin. After treatment with 0.5 µg/ml αHL or 10 ng/ml diphtheria toxin for 2 weeks, DNA was isolated and individual abundance of gRNAs compared to untreated CRISPR library-transduced cells was analyzed.

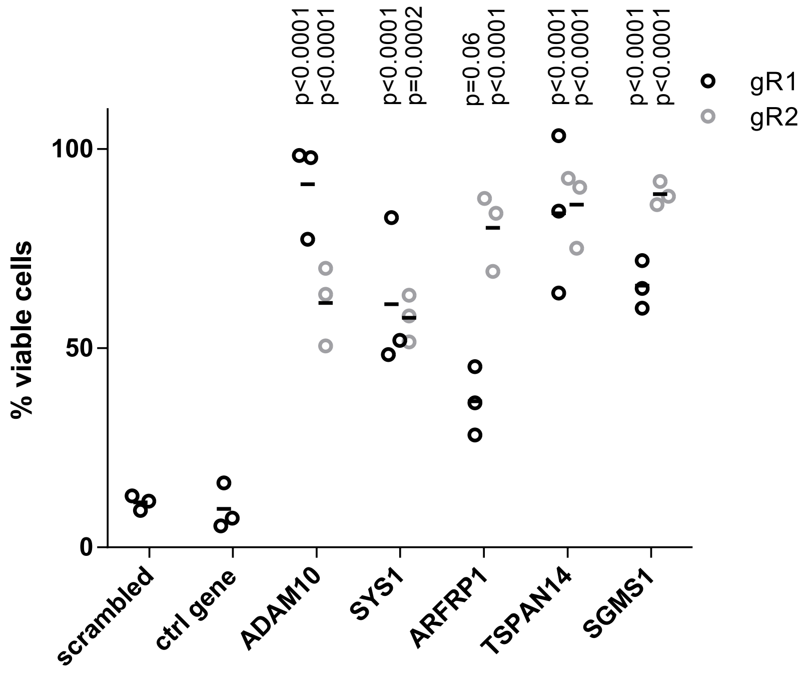

Supplementary Figure S3. Individually-targeted U937 are resistant to αHL

U937 cells transduced with Cas9 and two different gRNAs (gR1 & gR2) targeting the indicated genes were incubated with 1 µg/ml of αHL overnight. Percentage of viable cells was determined by flow cytometry and gating for DRAQ7 negative and forward side scatter characteristics as control U937 cells. As a control, a random scrambled and a gRNA targeting an unrelated control gene (Neutrophil Elastase, NE) were included. Each circle represents an independent experiment, line indicates mean value between the three experiments.

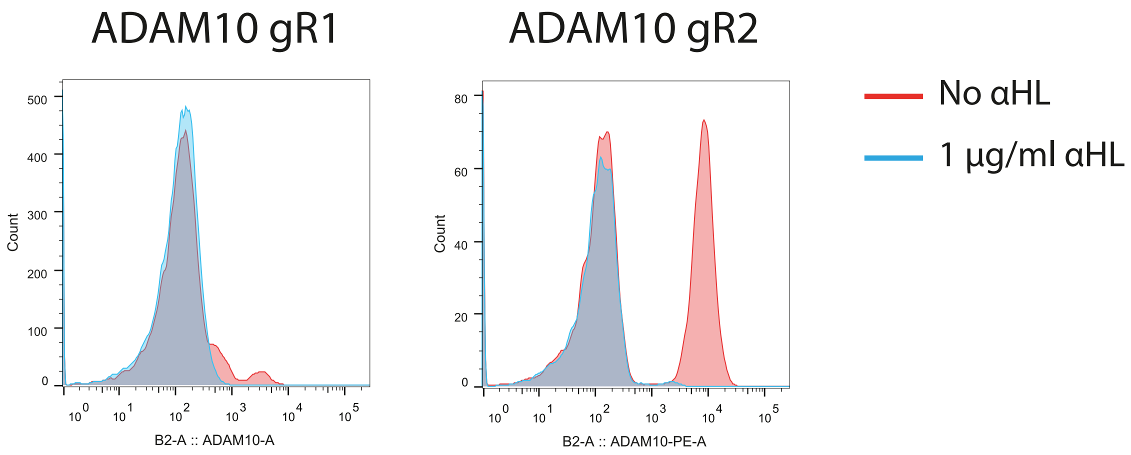

Supplementary Figure S4. Comparison survival ADAM10 gR1 versus gR2 after αHL treatment

U937 cells targeted with gR1 or gR2 were treated with 1 µg/ml αHL overnight. Histograms depict ADAM10 staining of viable cells and shows that efficiency of targeting varies between gR1 and gR2 with gR1 resulting in mostly ADAM10-negative cells whereas targeting with gR2 results in ADAM10-negative and ADAM10-positive cells.

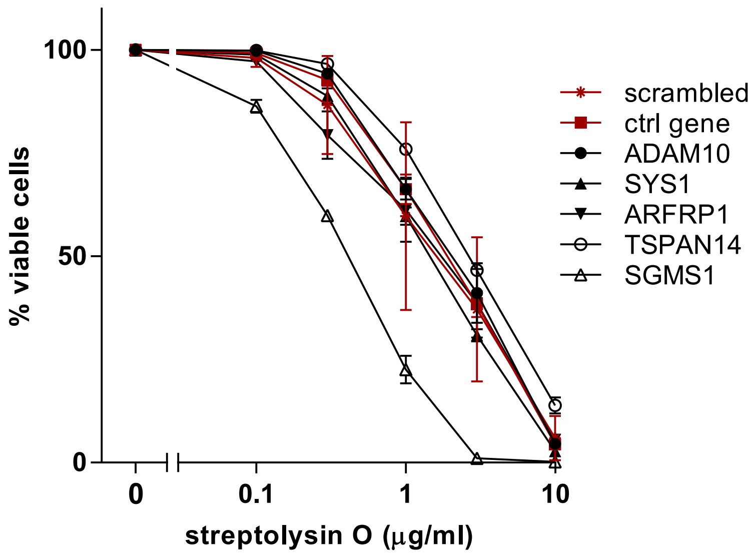

Supplementary Figure S5. Individually-targeted U937 are susceptible to SLO

Gene-targeted U937 cell clones were challenged with 0 - 10 µg/ml SLO for six hours, washed with PBS and stained with 0.6 µM DRAQ7. Percentage of viable cells was determined by flow cytometry and gating for DRAQ7 negative and forward side scatter characteristics as control U937 cells. Graph represents mean + SEM of three independent experiments.

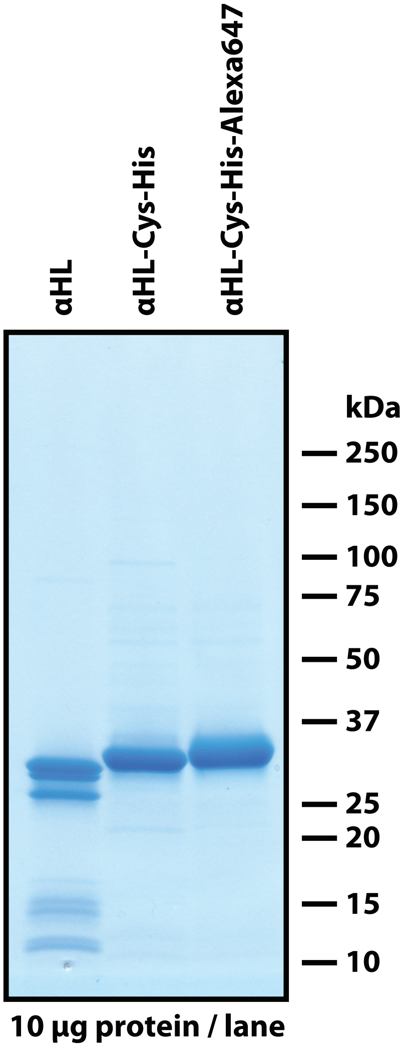

Supplementary Figure S6. αHL labeling

Purified αHL protein samples (unlabeled & labeled with Alexa Fluor 647) were separated by SDS-PAGE and stained with Coomassie blue.

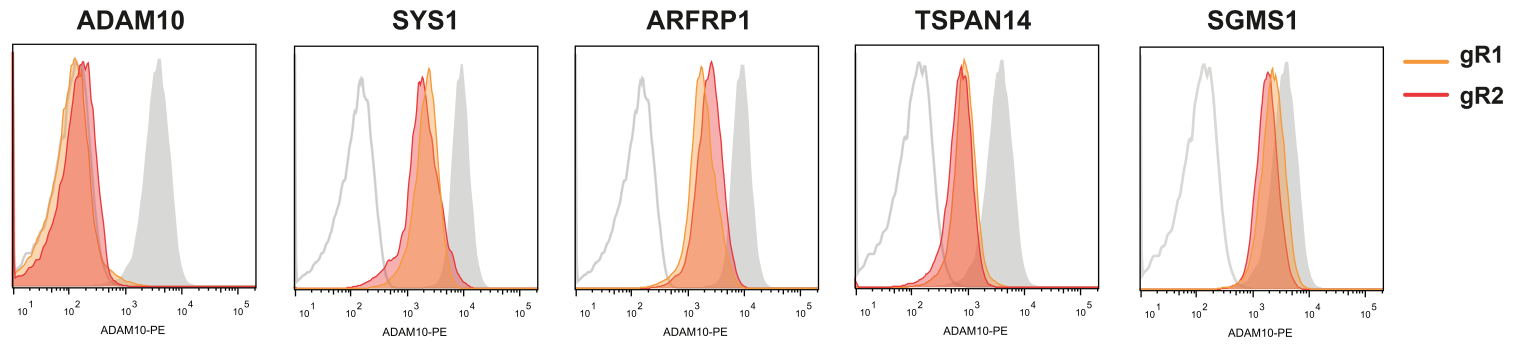

Supplementary Figure S7. ADAM10 expression of CRISPR-targeted U937 clones

U937 clones targeted with the gRNAs to the indicated genes were incubated with an ADAM10 antibody, followed by a PE-labeled secondary goat-anti-mouse antibody. A representative histogram for ADAM10 expression of two CRISPR-targeted clones for each gene is shown.

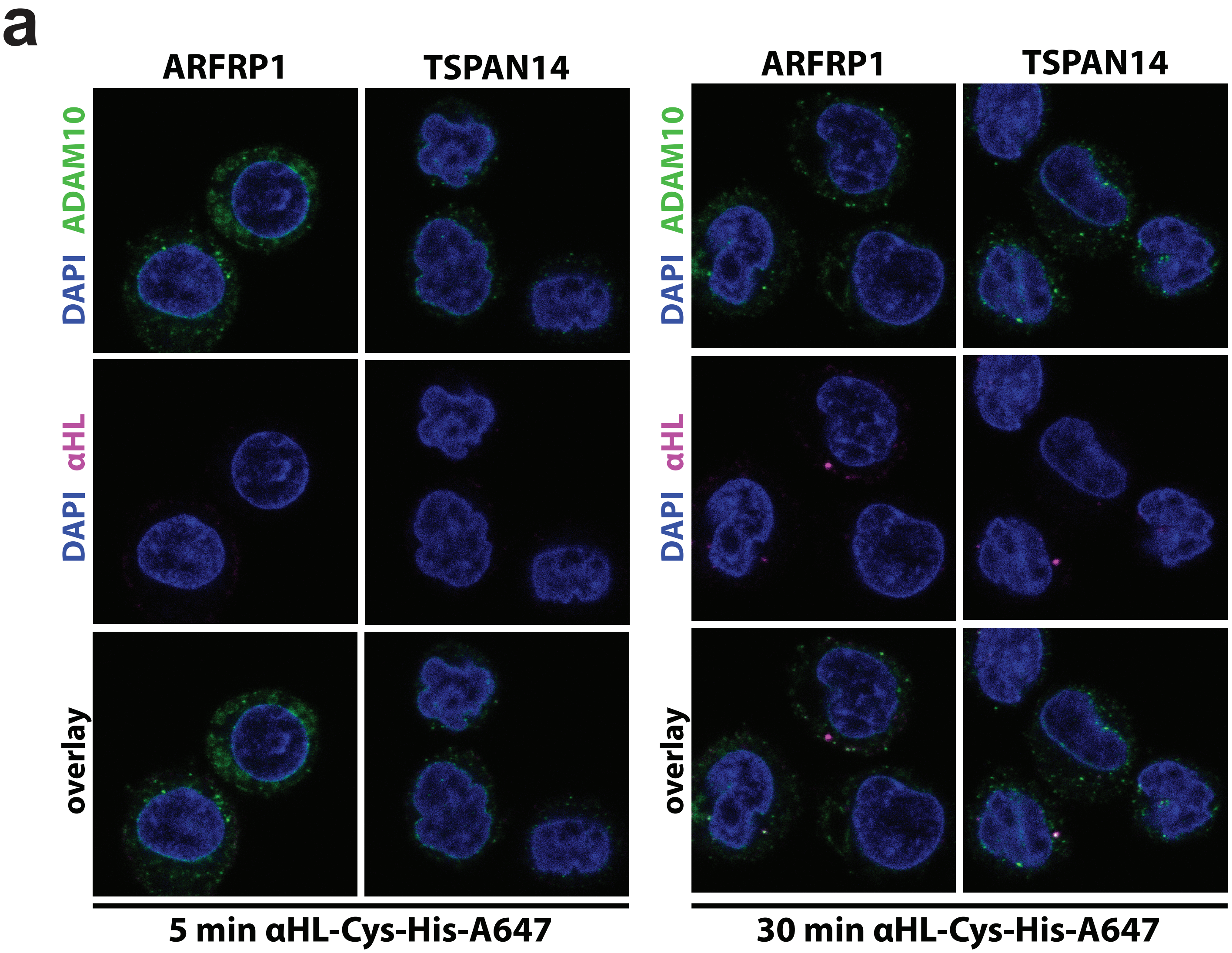

b

		ctrl gene	ADAM10	SYS1	ARFRP1	TSPAN14	SGMS1
5 min	Pearson (> threshold):	0.250	-0.650	-0.060	-0.300	-0.410	-0.490
	Manders tM1 (αHL):	0.692	0.117	0.665	0.506	0.340	0.259
	Manders tM2 (ADAM10):	0.417	0.063	0.334	0.215	0.143	0.100
30 min	Pearson (> threshold):	0.170	-0.110	0.130	0.020	-0.050	-0.230
	Manders tM1 (αHL):	0.666	0.140	0.702	0.551	0.568	0.446
	Manders tM2 (ADAM10):	0.400	0.082	0.295	0.281	0.180	0.170

* p-values according to Costes (100 iterations) for all analyses were 1.0

Supplementary Figure S8. ADAM10 and αHL localization upon intoxication

(a) U937 clones were intoxicated with 2 µg/ml αHL-Cys-His-Alexa647 for the indicated times, fixed, permeabilized and ADAM10 was visualized by staining with an anti-ADAM10 antibody (clone 11G2, magenta). Nuclei were stained with DAPI (blue). Representative confocal images of two independent experiments are shown.
(b) Entire z-planes of images were analyzed for colocalization. Pearson's and Manders' coefficients are depicted for each clone. Statistical significance was tested with 100 iterations according to Costes and p-values for all analyses were 1.

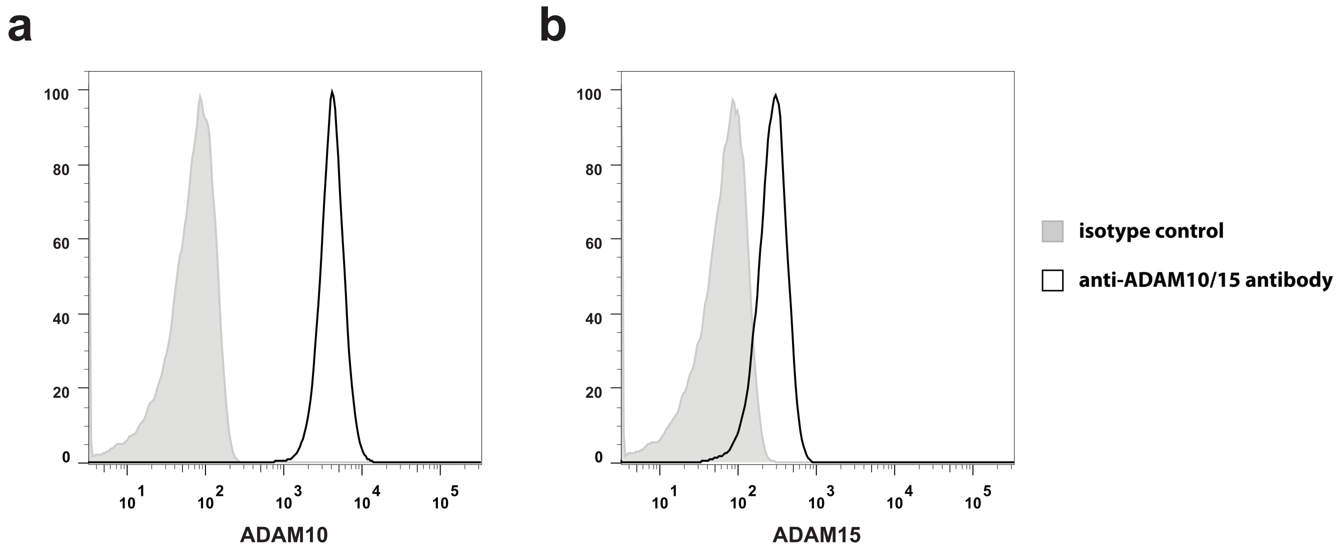

Supplementary Figure S9. ADAM10 and ADAM15 surface expression

(a) U937 cells were incubated with either 0.5 µg/ml IgG2b isotype or ADAM10 antibody for 30 minutes at 4 °C, washed with PBS, stained with a PE-labeled anti-mouse IgG for 30 minutes at 4 °C and analyzed by flow cytometry.
(b) Same experiment as in (a) only for IgG1 isotype and ADAM15 antibody.

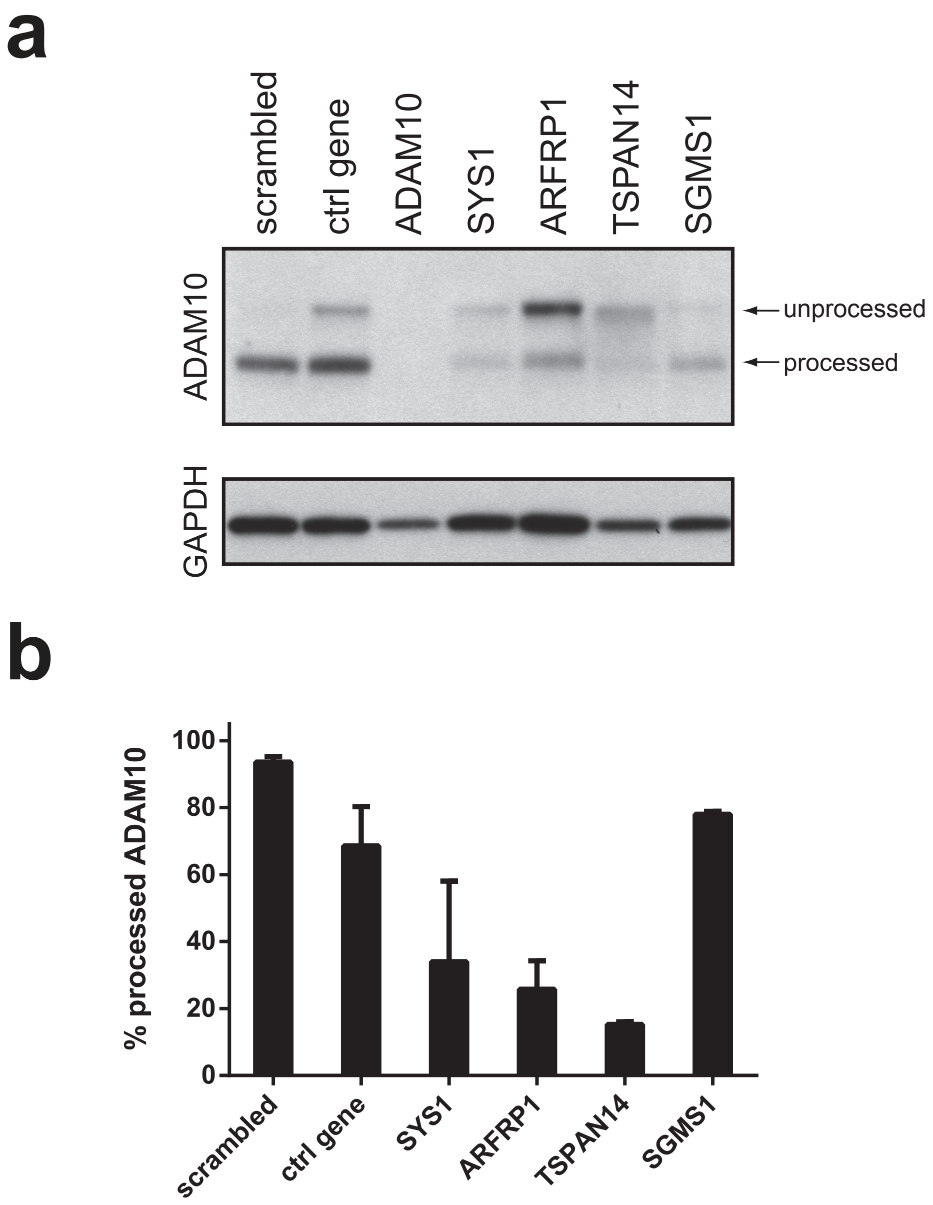

Supplementary Figure S10. Immunoblot analysis of ADAM10 processing in CRISPR-targeted clones

(a) U937 clones were lysed in non-reducing Laemmli buffer and proteins were separated on a non-reducing SDS-PAGE gel. Immunoblot analysis was performed using a monoclonal anti-ADAM10 antibody (clone 11G2) and anti-GAPDH antibody (clone 14C10). This blot is a representative of two independent experiments.
(b) The relative amount of processed ADAM10 compared to the total amount of ADAM10 was determined from western blots via densitometry. Graphs represent mean + SD from two independent experiments.

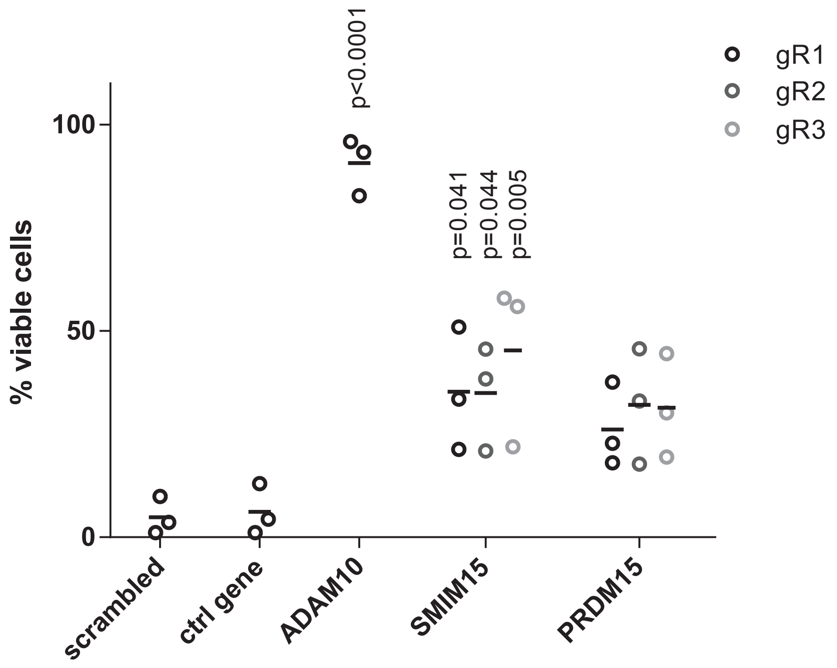

Supplementary Figure S11. SMIM15- and PRDM15-targeted cells are resistant to αHL

U937 cells transduced with Cas9 and up to three different gRNAs (gR1, gR2, gR3) targeting the indicated genes were incubated with 1 µg/ml of αHL overnight. Percentage of viable cells was determined by gating for DRAQ7 negative and forward side scatter characteristics as control U937 cells. As a control, a random scrambled and a gRNA targeting an unrelated control gene (Neutrophil Elastase, NE) were included. Each circle represents an independent experiment, line indicates mean value between the three experiments.
